# Vitamin D deficiency and prognostics among patients with pancreatic adenocarcinoma

**DOI:** 10.1186/1479-5876-11-206

**Published:** 2013-09-08

**Authors:** May Cho, Parvin F Peddi, Kevin Ding, Ling Chen, Denise Thomas, Jian Wang, Albert C Lockhart, Benjamin Tan, Andrea Wang-Gillam

**Affiliations:** 1 Department of Medicine, Washington University in St. Louis, St. Louis, MO, USA; 2 Division of Medical Oncology, Department of Medicine, Washington University School of Medicine, 660 S Euclid Ave, St. Louis, MO 63110, USA; 3 Saint Louis University, St. Louis, MO, USA; 4 Division of Biostatistics, Washington University in St. Louis, St. Louis, MO, USA; 5 Division of Hematology and Oncology, University of California, Los Angeles, CA, USA; 6 Division of Oncology, The Second Affiliated Hospital of Zhengzhou University, Zhengzhou, Henan, People’s Republic of China

**Keywords:** Vitamin D, Vitamin D receptor, Pancreatic cancer, Prognostic factors

## Abstract

**Background:**

The prevalence of vitamin D deficiency among patients with cancer has been previously reported. Because vitamin D is fat soluble, patients with pancreatic adenocarcinoma may have an especially high risk of vitamin D deficiency in association with ongoing and varying degrees of malabsorption. However, little is known about the correlation between vitamin D status and prognosis in these patients.

**Methods:**

We conducted a retrospective review of vitamin D status in patients with pancreatic adenocarcinoma who were treated at Siteman Cancer Center. Patients’ demographic information, clinical staging at the time of vitamin D assessment, vitamin D levels, and survival data were collected. Vitamin D deficiency was defined as a serum 25-hydroxyvitamin D (25[OH]D) level of less than 20 ng/mL, and vitamin D insufficiency was defined as a 25(OH)D level of between 20 ng/mL and 30 ng/mL.

**Results:**

Between December 2007 and June 2011, 178 patients with pancreatic adenocarcinoma had their vitamin D levels checked at the time of initial visit at this center. Of these 178 patients, 87 (49%) had vitamin D deficiency, and 44 (25%) had vitamin D insufficiency. The median 25(OH)D level was significantly lower among nonwhite patients and among patients with stage I and II disease. A 25(OH)D level of less than 20 ng/mL was found to be associated with poor prognosis (p = 0.0019) in patients with stage III and IV disease.

**Conclusions:**

Vitamin D insufficiency and deficiency were prevalent among patients with pancreatic adenocarcinoma. The vitamin D level appears to be prognostic for patients with advanced pancreatic adenocarcinoma, and its effects should be further examined in a prospective study.

## Introduction

Pancreatic cancer is the fourth leading cause of cancer-related death in the United States. In 2013, an estimated 45,220 new cases of pancreatic adenocarcinoma were diagnosed, and nearly 38,460 people died as a result of the disease [1]. Surgery is the only curative measure, but more than half of patients are not candidates for surgical resection at the time of diagnosis, and the 5-year survival rate is just 6% [1].

Vitamin D is a steroid hormone that plays an essential role in the maintenance of calcium, phosphate, and bone metabolism. Emerging evidence demonstrates that vitamin D has a plethora of antitumor properties as well, including the induction of cell differentiation, the stimulation of apoptosis, and the inhibition of cell proliferation, angiogenesis, and metastasis [2,3]. Ample epidemiologic studies have suggested that individuals with sufficient vitamin D levels are at lower risk for multiple types of cancers, including colorectal, breast, prostate, lung, and ovarian cancers [4]. For example, the risk of developing colorectal cancer was 50% lower among individuals with serum 25-hydroxyvitamin D (25[OH]D) levels of 33 ng/mL or more as compared with those with 25(OH)D levels of 12 ng/mL or less [5]. The risk of pancreatic cancer in association with vitamin D levels has been controversial; one study suggested an increased risk of cancer with 25(OH)D levels of more than 100 nmol/L [6], whereas another reported that the plasma level of 25(OH)D was inversely associated with the odds of developing pancreatic cancer [7]. A recent Canadian study suggested that the genes involved in the vitamin D pathway are associated with pancreatic cancer risk [8].

Vitamin D levels have also been shown to have an impact on the clinical outcomes of several cancer types, including breast, colon, lung, and prostate cancers as well as leukemia and lymphoma [9-13]. For example, higher 25(OH)D levels at the time of diagnosis in patients with colorectal cancer were associated with a significant reduction in overall mortality (p = 0.02) and an improvement in overall survival [10]. Patients with prostate cancer whose serum 25(OH)D levels were medium (50–80 nmol/L) or high (>80 nmol/L) had significantly better prognoses as compared with those with low (<50 nmol/L) serum concentrations [11]. Although studies have suggested that higher vitamin D levels are significantly associated with improved survival among patients with certain cancers, to date no study has examined the relationship of vitamin D with pancreatic cancer survival.

We hypothesized that there is a high incidence of vitamin D deficiency in the pancreatic cancer patient population, because these patients frequently have exocrine dysfunction. Furthermore, we hypothesized that low vitamin D levels may be associated with worse prognoses in this patient population as has been seen with other malignancies. In this article, we report our study of the prevalence of vitamin D deficiency among patients with pancreatic adenocarcinoma, and we evaluate the prognostic significance of vitamin D levels for this patient population.

## Materials and methods

We conducted a retrospective review of patients with pancreatic adenocarcinoma who were evaluated at Siteman Cancer Center (SCC) in St. Louis, Missouri, between December 2007 and June 2011. The retrospective registry was established to capture all patients after institutional review board approval was obtained. Patients with pancreatic adenocarcinoma who had at least one vitamin D level recorded at the time of diagnosis or the first time that they presented to the center were included in the study. Patients were only excluded if they did not have their vitamin D levels checked at their first presentation. Patients with stage I and II pancreatic cancer usually presented to the medical oncologists after surgery. Therefore, their first recorded vitamin D levels were after their surgeries and before their first chemotherapy treatments. The same chemiluminescent assays were used to measure patient vitamin D levels at this center throughout the study.

Patients’ demographic information, clinical stage at the time of vitamin D assessment, vitamin D levels, and clinical outcomes were collected directly from the institutional electronic medical record system. Vitamin D levels at the initial clinical visit and at later visits after supplementation were recorded. Chemotherapy treatments were given at the discretion of oncologists. Data regarding the treatments that patients received were not collected. Patients’ times of death were obtained from the electronic medical record and verified by the Social Security Death Index. In accordance with previous studies, vitamin D deficiency was defined as a serum 25(OH)D level of less than 20 ng/mL; vitamin D insufficiency was defined as a 25(OH)D level of 20 ng/mL to 30 ng/mL; and vitamin D sufficiency was defined as a 25(OH)D level of at least 30 ng/mL [3,14]. Patients’ vitamin D levels at the time of initial visit to SCC were used for prognostic studies.

Comparisons of patients’ mean vitamin D levels with respect to baseline characteristics were carried out with the use of the Student’s *t*-test. Demographic and clinical characteristics among the three groups with regard to vitamin D status (i.e., deficient, insufficient, or sufficient) were compared via the chi-square test. Overall survival was defined as the time from pathological diagnosis to the time of death from any cause by June 2011. Survivors were censored at the date of last contact. The time to death in each vitamin D group (i.e., a 25[OH]D level of ≥20 ng/mL or <20 ng/mL) was described by the Kaplan-Meier product limit method, and the between-group differences were compared with the use of the log-rank test. Univariate and multivariate analyses were performed for patients with stage III and IV pancreatic cancer with the use of the Cox proportional hazards model. All analyses were conducted with a two-sided test at a significance level of 0.05. Statistical analyses were performed with SAS 9.2 software (SAS Institute, Cary, North Carolina).

## Results

### Patient characteristics

Of the 178 patients included in this study, 161 (90%) were 50 years old or older. The majority of the patients (87%) were Caucasian. The sample population was nearly equally divided between genders, with 82 (46%) being female and 96 (54%) being male. Most patients (64%) had advanced disease (i.e., stage III and IV). The majority of patients in this study had body mass indices of more than 25 kg/m2.

### Vitamin D levels

Vitamin D status was analyzed across several variables: age, race, gender, tumor stage, season in which vitamin D was drawn, and BMI (Table 1). There were no statistically significant differences in mean 25(OH)D levels between groups with regard to age, gender, season, or BMI. Lower mean 25(OH)D levels were seen in non-white patients as compared with white patients (13.95 vs. 23 ng/mL; p = 0.0007). Interestingly, patients with stage I and II disease appeared to have lower 25(OH)D levels than patients with advanced stage III and IV disease (19.33 vs. 23.71 ng/mL; p = 0.0149). There was a trend toward higher vitamin D levels during summer and autumn; however, the difference was not statistically significant.

**Table 1:** Patient characteristics

	**Vitamin D level (ng/mL)**			
**Patients**	**No. (%)**	**Mean**	**Standard deviation**	**P value**
**Age:**				
<50 years	17 (10)	20.59	8.70	
≥50 years	161 (90)	22.30	12.98	0.4731
**Race:**				
White	155 (87)	23.00	12.44	
Non-white	23 (13)	13.95	10.89	0.0007
**Gender:**				
Female	82 (46)	22.17	12.83	
Male	96 (54)	22.10	12.52	0.9722
**Tumor stage:**				
I and II	64 (36)	19.33	9.85	
III and IV	114 (64)	23.71	13.74	0.0149
**Season:**				
Autumn	28 (16)	23.57	13.80	
Winter	56 (31)	21.18	11.80	
Spring	60 (34)	21.12	12.61	
Summer	34 (19)	24.32	13.17	0.5566
**Body mass index:**				
<25 kg/m^2^	68 (38)	22.06	11.43	
≥25 kg/m^2^	110 (62)	22.18	13.37	0.1659

Of the 178 patients in the study, 87 (49%) had vitamin D deficiency, and 44 (25%) had vitamin D insufficiency (Table 2). Only 47 (26%) of the patients had sufficient vitamin D levels. Vitamin D sufficiency was much lower among non-white patients (12%) than white patients (29%). Only 13 patients (20%) with stage I and II pancreatic adenocarcinoma had sufficient vitamin D levels, whereas 34 patients (30%) with stage III and IV disease had sufficient levels of vitamin D.

**Table 2:** Distribution of vitamin D status

	**Deficient vitamin D**	**Insufficient vitamin D**	**Sufficient vitamin D**	**P value**
	**(<20 ng/mL)**	**(20–29 ng/mL)**	**(≥30 ng/mL)**	
	**No. (%)**	**No. (%)**	**No. (%)**	
**All patients**	87 (49)	44 (25)	47 (26)	
**Age:**				
<50 years	6 (35)	9 (53)	2 (12)	
≥50 years	81 (50)	35 (22)	45 (28)	0.29
**Race:**				
White	70 (45)	40 (26)	45 (29)	
Non-white	17 (74)	4 (17)	2 (9)	0.029
**Gender:**				
Female	38 (46)	22 (27)	22 (27)	0.79
Male	49 (51)	22 (23)	25 (26)	
**Tumor stage:**				
I and II	36 (56)	15 (23)	13 (20)	
III and IV	51 (45)	29 (25)	34 (30)	0.27
**Season:**				
Autumn	11 (40)	9 (32)	8 (28)	
Winter	31 (55)	10 (18)	15 (27)	
Spring	33 (55)	14 (23)	13 (22)	
Summer	12 (35)	11 (32)	11 (32)	0.39
**Body mass index:**				
<25 kg/m^2^	30 (44)	19 (28)	19 (28)	
≥25 kg/m^2^	57 (52)	25 (23)	28 (25)	0.058

A subset of 71 patients with vitamin D deficiency or insufficiency was supplemented with 50,000 units of vitamin D weekly for 10 to 12 weeks. Vitamin D levels were reassessed after supplementation. Forty-two of these patients (55%) had not achieved sufficient vitamin D levels at the time of reassessment, despite supplementation. The mean vitamin D level was 22 ng/mL even after the course of vitamin D supplementation.

### Prognostic significance

**Figure 1:**
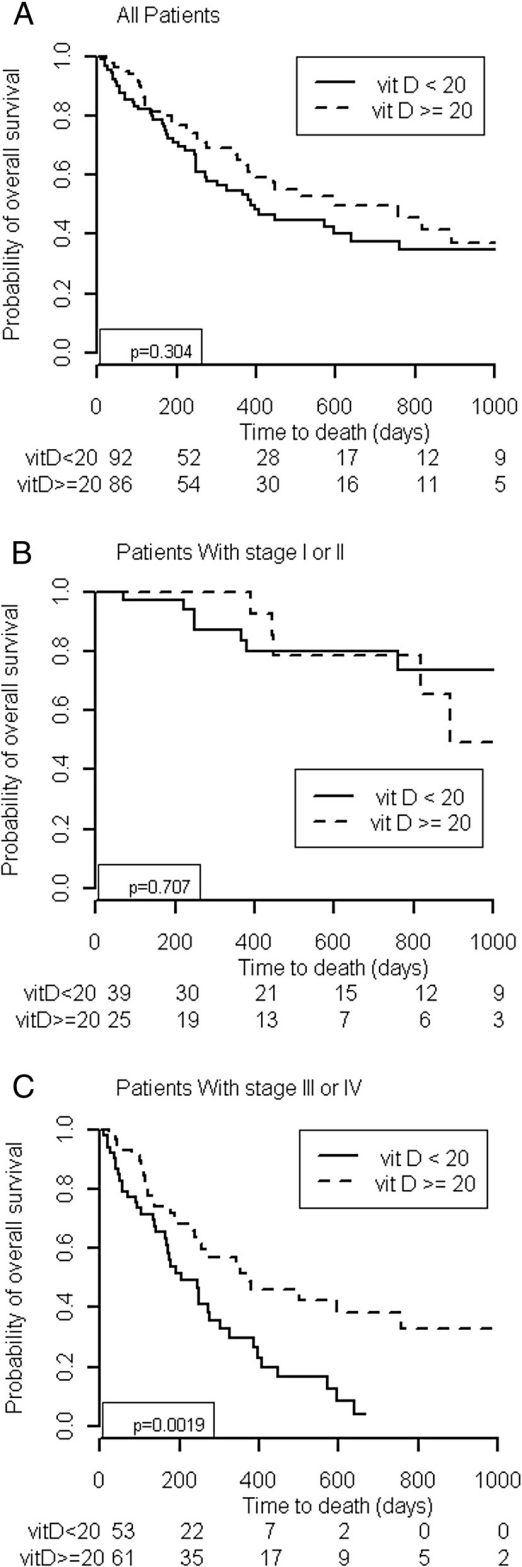
Overall survival Kaplan-Meier plot for patients with 25-hydroxyvitamin D levels of less than 20 ng/mL and more than 20 ng/mL with all stages of pancreatic cancer (A), with stage I or II pancreatic cancer (B), and with stage III or IV pancreatic cancer (C).

Figure 1 shows the overall correlation between the survival of patients with pancreatic cancer and their vitamin D levels at the time of initial visit to SCC. Of 178 patients enrolled in this study, 82 died, and 96 were censored. The median overall length of survival was 447 days. For patients with all stages combined or stage I and II diease, 25(OH)D levels of less than 20 ng/mL did not affect overall survival. Vitamin D levels of less than 20 ng/mL appeared to be associated with a worse prognosis among patients with stage III and IV disease (p = 0.0019). The multivariate analysis of patients with stage III and IV disease showed that lower vitamin D levels and lower BMIs had prognostic significance shown in Table 3.

**Table 3:** Univariate and multivariate cox regression analyses of different prognostic variables for overall survival in patients with stage III and IV of pancreatic cancer

		**Univariate analysis**		**Multivariate analysis**	
	**No. of patients**	**P Value**	**Regression coefficients (Standard error)**	**Hazard ratio (95% confidence interval)**	**P value**
**Age:**					
≥50 years	103	0.7990	0.109 (0.428)	1.101 (0.450-2.693)	0.8334
<50 years	11				
**Race:**					
Non-white	17	0.7842	0.095 (0.345)	1.020 (0.504-2.064)	0.9556
White	97				
**Gender:**					0.2731
Female	52	0.1755	−0.334 (0.246)	0.751 (0.450-1.253)	
Male	62				
**Vitamin D level:**					
<20 ng/mL	53	0.0024	0.752 (0.248)	1.991 (1.156 - 3.430)	0.0131
≥20 ng/mL	61				
**Season:**					
Autumn	19	0.5497	−0.436 (0.375)	0.648 (0.293-1.433)	0.1789
Spring	38		0.017 (0.303)	0.997 (0.549-1.812)	
Summer	22		0.142 (0.335)	1.672 (0.808-3.459)	
Winter	35				
**Body mass index:**					
≥25 kg/m^2^	72	0.0040	0.142 (0.335)	0.441 (0.265-0.733)	0.0016
<25 kg/m^2^	42				

## Discussion

Epidemiological studies have suggested a correlation between vitamin D status and an individual’s risk for pancreatic cancer [7]. There is a high prevalence of vitamin D deficiency in the general population (41.6%) [15] and among patients with other malignancies [16]. In our single-institution study, the prevalence of vitamin D deficiency and insufficiency was higher than that observed in the general population and occurred in 74% of the 178 patients studied. Klapdor and colleagues reported an even higher rate of 92.4% of 103 patients with pancreatic cancer who had 25(OH)D levels of less than 30 ng/mL [17]. More than 90% of our non-white pancreatic cancer patients had vitamin D deficiencies; this was consistent with the findings of other studies that also reported a high prevalence of vitamin D deficiency among non-whites.

To our knowledge, none of the prior studies assessed the correlation between pancreatic cancer stage and vitamin D status. Surprisingly, our study demonstrated that patients with stage I and II disease had lower vitamin D levels than patients with stage III and IV disease. Most of the patients with stage I and II disease were referred to medical oncologists after their surgeries; thus, their vitamin D levels were first checked after their operations. Among patients undergoing pancreatectomy, vitamin D levels are thought to be lower as a result of the surgical procedure itself in addition to associated exocrine dysfunction [18]. This idea is in alignment with the reported high rate of vitamin D deficiency found among patients after resection. The season in which the vitamin D level was obtained did not have a statistical impact, although higher levels were observed during summer and autumn. Although some studies have stated that patients with higher BMIs have lower vitamin D levels [19,20], this was not observed in the current study. Patients with pancreatic cancer have lower vitamin D levels, regardless of the season or their BMI. Malabsorption and disease burden have more of an impact on vitamin D levels in these patients than synthetic insufficiency from the sunlight or storage capacities.

Our study further evaluated the association between baseline vitamin D levels and the prognoses of patients with pancreatic cancer. The association of the vitamin D level with the prognoses of various cancer populations has been variable in the literature. Although a sufficient vitamin D level was associated with a better prognosis in patients with colon cancer [10,21] and a lower disease relapse and death rate in patients with melanoma [22], the prognostic value of the vitamin D level was not statistically significant in patients with non–small-cell lung cancer [23], and it was controversial in patients with breast cancer [24,25]. We did not find an association between vitamin D deficiency and pancreatic cancer prognosis when all stages were combined. However, to our knowledge, this study is the first to suggest a significantly worse overall survival rate for patients with vitamin D levels of less than 20 ng/mL with stage III or IV pancreatic cancer. Multivariate analysis also confirms that the vitamin D level has an impact on the prognosis of pancreatic cancer. Although patients with stage I and II pancreatic cancer have lower vitamin D levels than those with stage III and IV, there is no significant association with poorer outcomes. The prognostic significance of vitamin D levels in patients with stage III and IV pancreatic cancer should be further examined in a prospective study.

The expression of the vitamin D receptor (VDR) and its polymorphisms modulate the activity of vitamin D, which may in turn affect the prognosis of patients with pancreatic cancer. Certain VDR polymorphisms show prognostic significance for melanoma, squamous cell cancer of the head and neck, and non–small-cell lung cancer [23,26]. In a recent genome-wide association study of the overall survival of patients with pancreatic cancer, VDR gene polymorphism was associated with several prognostic factors [27]. Because patients with pancreatic cancer have a high prevalence of vitamin D deficiency in general, patients with lower VDR expression would have poorer prognoses as well as poorer responses to chemotherapy as a result of their vitamin D deficiencies. Our study was retrospective in nature and, unfortunately, patient VDR levels are not routinely checked, so we could not include VDR levels in our analysis. However, the future prospective examination of VDR levels in patients with pancreatic cancer is definitely warranted.

Our study suggested that the routine vitamin D supplementation of 50,000 International Units weekly for a short period of time (i.e., 10–12 weeks) may not be adequate to normalize vitamin D levels in most patients with pancreatic cancer. More prolonged supplementation may be required to achieve adequate levels. Exocrine pancreatic insufficiency may add another layer of complexity, thereby limiting sufficient supplementation with standard dosing. One study used a higher dose of vitamin D supplementation of up to 20,000 International Units daily for individuals with severe pancreatic exocrine insufficiency [17] and reported the normalization of vitamin D levels by individually adjusting oral vitamin D intake. The exact dose of supplementation may vary with the degree of insufficiency and the potential correction of the insufficiency with pancreatic enzymes. Regardless, the clinical impact of the normalization of vitamin D levels on disease progression and patient survival is still unknown and will require future prospective trials.

The current study is limited by its retrospective nature, the lack of accurate documentation of vitamin D supplementation before the diagnosis of pancreatic cancer, and the lack of control over vitamin D supplementation regimens. This was a single-institution study, so our cohort may differ from other pancreatic cancer populations. The study also demonstrates selection bias in that sicker patients were probably referred to our tertiary cancer center. The study attempted to not further limit the pool of patients by allowing all patients with baseline vitamin D level measurements to be included. Systemic treatment of patients was not controlled in this study but that would not be expected to bias the results in a particular way.

In summary, the current report confirms the high prevalence of vitamin D deficiency and insufficiency among patients with pancreatic cancer. Patients with early-stage pancreatic cancer have lower vitamin D levels as compared with those patients with advanced disease. However, only in patients with stage III and IV pancreatic cancer was there a significant association with poorer outcomes. We recommend the systematic screening of patients with pancreatic cancer for vitamin D deficiency and the rigorous monitoring of these patients while they are receiving oral supplementation. Further prospective investigations should be conducted to study vitamin D supplementation for patients with pancreatic cancer and to determine the prognostic value of vitamin D levels and VDR expression in this patient population.

## Abbreviations

25(OH)D: 25-Hydroxyvitamin D; BMI: Body mass index; VDR: Vitamin D receptor.

## Competing interests

The authors declare that they have no competing interests.

## Authors’ contributions

AWG, BT, and ACL designed the study, recruited patients, and edited the manuscript. KD, DT, and JW helped with the collection of data. LC helped with statistics. PFP helped with analyzing data and editing the manuscript. MC collected and analyzed data and wrote the manuscript. All authors read and approved the final manuscript.
